# Cytomegalovirus lumbosacral polyradiculitis in patients with long-term use of an oral corticosteroid: a case report

**DOI:** 10.1186/s12883-022-02623-3

**Published:** 2022-03-14

**Authors:** Jung Hae Yun, Ming-Yen Hsiao, Mathieu Boudier-Revéret, Min Cheol Chang

**Affiliations:** 1 Department of Rehabilitation Medicine, College of Medicine, Yeungnam University, 317-1, Daemyungdong, Namku, Daegu, 705-717 Republic of Korea; 2 Department of Physical Medicine and Rehabilitation, College of Medicine, National Taiwan University, Taipei, Taiwan; 3 Department of Physical Medicine and Rehabilitation, National Taiwan University Hospital, Taipei, Taiwan; 4 Department of Physical Medicine and Rehabilitation, Centre hospitalier de l’Université de, Montréal, Montreal, Canada

**Keywords:** Cytomegalovirus, Viral infection, Radiculitis, Corticosteroid

## Abstract

**Background:**

The long-term use of an oral corticosteroid suppresses immunity. Here, we describe a case involving a patient with weakness in the bilateral lower extremities due to cytomegalovirus (CMV) lumbosacral polyradiculitis.

**Case presentation:**

A 64-year-old man visited a university hospital for symmetric motor weakness in both lower extremities (Medical Research Council grade: 2). Symptoms started 1 month before and gradually aggravated. The patient had been taking oral prednisolone for 10 years in order to control pain in multiple joints due to seronegative rheumatoid arthritis. He also had neuropathic pain on the entire right lower extremity and voiding difficulty. Gadolinium-enhanced magnetic resonance imaging revealed enhancement along the entire lumbosacral nerve roots. In the cerebrospinal fluid analysis (CSF), elevated white blood cell (WBC) count (19 cells/μL) and protein level (142.5 mg/dL) were observed. CMV detection by polymerase chain reaction (PCR) was positive. We diagnosed the patient as having lumbosacral polyradiculitis due to CMV. Ganciclovir (250 mg twice daily) was administered intravenously. Two months after initiating Ganciclovir, in the CSF analysis, CM detection by PCR was negative, and no WBC was found.

**Conclusion:**

We reported a patient who had symmetric motor weakness in the bilateral lower extremities induced by CMV lumbosacral polyradiculitis. Its occurrence seems to be related to immunosuppresion due to the long-term use of an oral corticosteroid. When a patient who is taking an oral corticosteroid shows motor weakness in the bilateral lower extremities, CMV lumbosacral polyradiculitis is one of the possible disorders to be differentiated.

**Supplementary Information:**

The online version contains supplementary material available at 10.1186/s12883-022-02623-3.

## Background

Cytomegalovirus (CMV) is a member of the herpesvirus family. CMV infection usually occurs early in life and is transmitted by close contact with infected body fluid [1]. After primary CMV infection, CMV remains latent with periodical reactivation [1]. In persons who are immunocompetent, primary CMV infection is usually asymptomatic or mild. However, in patients who are immunocompromised, distinctive symptoms with multi-organ involvement can be manifested by primary or recurrent CMV infections and can contribute significantly to morbidity and mortality [2, 3]. Occasionally, due to CMV radiculitis or myelitis, various neurological symptoms, such as motor weakness, sensory deficit, pain, and voiding difficulty may develop [4, 5]. Previously, some cases with CMV radiculitis or myelitis were reported in patients with human immunodeficiency virus (HIV) infection and patients with cancer who were receiving chemotherapy [4–6].

Corticosteroids have an inhibiting effect on a broad range of immune and inflammatory responses [7]. They are widely prescribed to patients with inflammatory and autoimmune disorders. They can also be used as an immunosuppressive drug following an organ transplant. Approximately 1% of the total adult population is known to receive oral corticosteroid therapy [8]. However, their long-term use can cause various side effects, including metabolic disease, osteoporosis, and cardiovascular diseases [8]. In addition, they can suppress immunity by interfering with the signals of the key inflammatory transcriptional regulators. Accordingly, patients who are taking an oral corticosteroids on the long-term have a high chance of being infected by bacteria, viruses, or fungi [9].

In the current study, we report an occurrence of CMV lumbosacral polyradiculitis in a patient with long-term use of oral corticosteroids.

## Case presentation

A 64-year-old man visited a university hospital for motor weakness in both lower extremities. The weakness developed suddenly without any prodromal illness 3 weeks prior to the hospital visit and gradually worsened. For controlling chronic pain in multiple joints due to seronegative rheumatoid arthritis, he had been taking oral prednisolone (4 mg per day, 2 mg bid) for 10 years, which was prescribed by the local pain clinic. The clinician in the local pain clinic used oral prednisolone because the patient’s pain was refractory to commonly used pain medications, including non-steroidal anti-inflammatory drugs, tramadol, and disease-modifying antirheumatic drugs (hydroxychloroquine and methotrexate). On physical examination, symmetrical motor weakness was observed in the bilateral lower extremities. The strengths of the bilateral hip flexor, knee extensor, ankle dorsiflexor, first toe extensor, and ankle plantar flexor were Medical Research Council (MRC) grade 2. He could not stand or walk. Any sensory deficit was not checked, but he had neuropathic pain on the entire right lower extremity (numeric rating scale: 4; 0 indicates no pain, and 10 indicates the most severe pain). Deep tendon reflexes of both the lower extremities were decreased. Ankle clonus and flexor plantar reflex were not present. He also had voiding difficulty. After self-voiding of 100 mL urine, residual urine was approximately 400 mL.

We admitted the patient, and laboratory tests were performed upon admission. White blood cell count (15,170 cells/μL) and C-reactive protein (2.011 mg/dL) were increased. Rheumatoid factor, anti-cyclic citrullinated peptides, anti Sjiogren’s syndrome (anti-Lo), and anti-rheumatoid arthritis (anti-Ra) antibodies, complement C3, and C4 were negative.

In the nerve conduction tests performed the next day (approximately 3 weeks after onset of symptoms), decreased amplitude was observed in the compound motor action potentials of the bilateral peroneal nerves recorded at extensor digitorum brevis (Rt: 0.3 mV, Lt: 0.4 mV) (Supplementary 1). This was also observed in bilateral tibial nerves recorded at abductor hallucis (Rt: 4.7 mV, Lt: 4.7 mV). Decreased sensory nerve action potentials in the bilateral superficial peroneal nerves (Rt: 4 μV, Lt: 9 μV) were also noted. A positive sharp wave (1+) was present at the right tibialis anterior, peroneus longus, and lumbar paraspinal muscles. The F-waves of the peroneal nerves bilaterally and H-reflexes from the tibial nerves bilaterally showed no response. The central motor conduction time to the bicep brachii, abductor pollicis brevis, and tibialis anterior, induced by transcranial magnetic stimulation, were normal. Furthermore, sensory evoked potentials from both median and posterior tibial nerves were within normal limits.

**Fig. 1:**
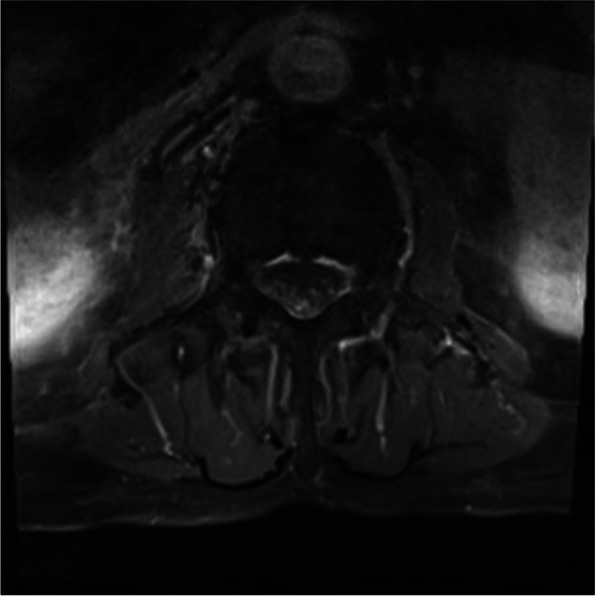
T1-weighted gadolinium-enhanced axial MRI at L4–5 level performed 3 weeks after the onset of symptoms due to cytomegalovirus polyradiculitis showing the enhancement in the entire lumbosacral nerve root

Magnetic resonance imaging (MRI) of the brain showed no abnormalities. The complete spine MRI showed central canal stenosis at C4–5 and C5–6 without significant cord compression and mild central canal stenosis at L5-S1. The gadolinium enhanced MRI revealed the enhancement along the entire lumbosacral nerve root (cauda equina) (Fig. 1). Cerebrospinal fluid (CSF) analysis was performed 1 month after the onset of symptoms (7th admission day): elevated white blood cell count (19 cells/μL: polymorphonuclear cell 25%, lymphocyte 75%) and protein level (142.5 mg/dL) were observed. Red blood cell was not present, and glucose level (70 mg/dL) was within the normal range. Bacterial culture, tuberculosis polymerase chain reaction (PCR) test, and Varicella-zoster virus IgG and IgM of the CSF were negative. However, CMV detection by PCR was positive. In the blood test, CMV detection by real time PCR showed 6.73 × 10^2^ IU/mL and 740 copies/mL. Human immunodeficiency virus antigen, antibody, and ribonucleic acid tests were negative. Therefore, the diagnosis of lumbosacral polyradiculitis due to CMV was confirmed. Ganciclovir (250 mg twice daily) was administered intravenously.

The nerve conduction test was conducted 2 months after the onset of symptoms. The compound motor action potentials of the bilateral peroneal nerve, recorded at extensor digitorum brevis, (Rt: 0.4 mV, Lt: 0.4 mV) remained similar to those noted in previous studies (Supplementary 1). However, those of the tibial nerves, recorded at abductor hallucis (Rt: 4.4 mV, Lt: 2.6 mV), were decreased. Additionally, in the electromyographic test, a positive sharp wave (2 + ~ 3+) was present at all the evaluated muscles of the lower extremities, including the iliopsoas, vastus medialis, tibialis anterior, peroneus longus, tensor fascia latae, gastrocnemius, gluteus maximus, and the lumbar paraspinal muscles. The results of the sensory nerve action potentials were not changed compared to those of the prior test. These findings were indicative of lumbosacral polyradiculopathy.

Three months after the onset of symptoms, in the CSF analysis, CMV detection by PCR was negative and no WBC was found. CMV detection by PCR in blood test was also negative. We stopped the administration of Ganciclovir. Four months after the onset of symptoms, the strength in the lower extremities were examined, and MRC grade 3 on the right side and MRC grade 4 on the left side. The neuropathic pain on the right lower extremity had completely disappeared. Additionally, voiding difficulty recovered completely. The patient provided informed signed consent for participation in the study.

## Discussion and conclusions

Here, we report the occurrence of CMV lumbosacral polyradiculitis in a patient with long-term use of oral corticosteroid which seemed to have weakened the patient’s immunity and have made him vulnerable to CMV infection [8, 9]. CMV lumbosacral polyradiculitis is known to usually develop in patients with HIV infection, in which most of the cases of CMV polyradiculitis are reported in patients with HIV infection [4, 5, 10]. In our case report, we found that long-term use of oral corticosteroid might be a risk factor of CMV lumbosacral polyradiculitis.

In a patient with sudden-onset symmetric motor weakness in the lower extremities, clinicians should consider several differential diagnoses, such as spinal disorders (spondylotic myelopathy, transverse myelitis, and spinal cord infarct), early-stage Guillain-Barre syndrome, lumbosacral radiculitis due to viral or bacterial infection, and myositis. Clinicians should consider the likelihood of each disorder mentioned prior and determine which of these pathologies is the one in question using clinical history, physical examination, imaging modalities, and electrodiagnostic study.

In our patient, gadolinium-enhanced MRI showed inflammation (nerve enhancement) in multiple lumbrosacral nerve roots, which was a supportive finding in the diagnosis of CMV lumbosacral polyradiculitis. Nerve enhancement in gadolinium-enhanced MRI is related to the accumulation of gadolinium in granulation tissue, inflammatory cytokines, and disruption of endoneurial capillaries [11]. Cytokines induced by CMV infection result in the breakdown of the blood-nerve barrier and increase vascular permeability, which causes the enhancement in the nerve tissue. Other than virus infection, the enhancement of multiple nerve roots can be present in radiculitis due to bacterial infection, Guillain-Barre syndrome, and neuralgic amyotrophy [11–13].

There remains a lack of data for determining the therapeutic outcomes of CMV polyradiculitis. Almost all previously reported cases of CMV polyradiculitis occurred in patients with HIV; however, many patients died despite administration of Ganciclovir [4, 5, 10]. However, we cannot know whether patients died from acquired immune deficiency syndrome or CMV infection. To clarify the prognosis of CMV radiculitis, further cases or studies should be reported or conducted.

Ganciclovir is the first-line of treatment for CMV polyradiculitis. Acyclovir is not used in treating CMV polyradiculitis due to inferior response compared with Ganciclovir [14]. After finishing treatment with Ganciclovir (when CMV PCR in the CSF analysis is negative), Valacyclovir can be applied to prevent recurrence [14].

Most previous case reports of polyradiculopathy due to CMV infection were reported in immunodeficient patients. These individuals had HIV infection or underwent chemotherapy that resulted in poor outcomes [4–6]. In the current study, we reported a patient with symmetric motor weakness in the lower extremities due to CMV lumbosacral polyradiculitis. We believe that the development of polyradiculitis due to CMV infection was associated with the long-term use of an oral corticosteroid. This case report is unique as it is the first to study steroid discontinuation in combination with intravenous Ganciclovir treatment (after oral steroid treatment) in CMV polyradiculitis, in a seronegative rheumatoid arthritis patient. Because severe damage of the nerve roots can lead to poor therapeutic outcomes, it should be rapidly diagnosed, and treatment with Ganciclovir should be promptly administrated.

## Supplementary Information

**Additional file 1.**

## Data Availability

All data generated or analyzed during this study are included in this published article.

## Abbreviations

CMV: Cytomegalovirus
MRI: Magnetic resonance imaging
