# Supplementary material for: Cytomegalovirus lumbosacral polyradiculitis in patients with long-term use of an oral corticosteroid: a case report
Source: BMC Neurol. 2022 Mar 14;22:90. doi: 10.1186/s12883-022-02623-3 (PMC8919595; doi:10.1186/s12883-022-02623-3)
Supplement: Supplementary file 1 — Additional file 1. [file 12883_2022_2623_MOESM1_ESM.docx]

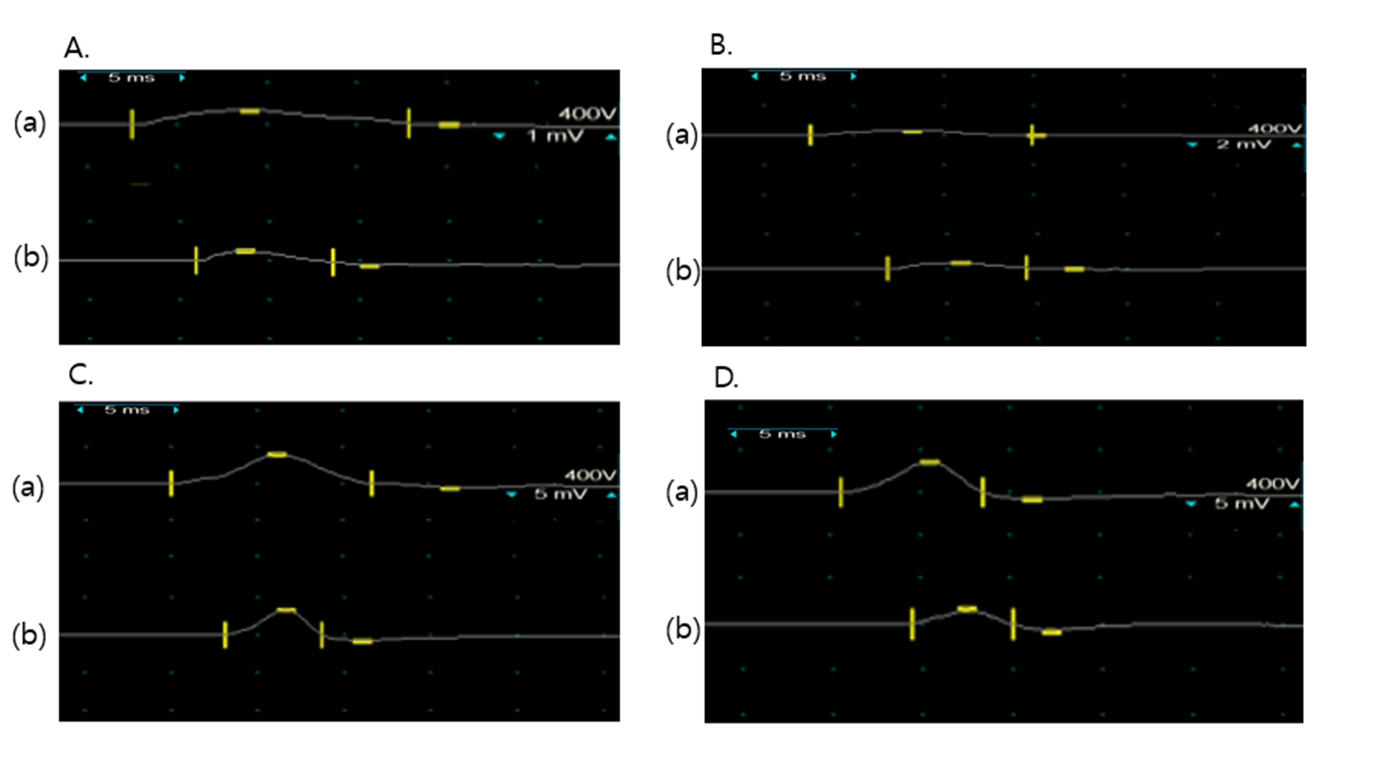


Compound motor action potential test; A. Rt. Peroneal nerve, B. Lt. peroneal nerve, C. Rt. Tibial nerve, D. Lt. Tibial nerve; (a) three weeks after onset of symptoms, (b) two months after onset of symptoms
